# Methyl­(phenyl)­bis­(quinoline-2-carbox­ylato-κ^2^ *N*,*O*)tin(IV) monohydrate

**DOI:** 10.1107/S1600536810054437

**Published:** 2011-01-12

**Authors:** Marzieh Vafaee, Mostafa M. Amini, Seik Weng Ng

**Affiliations:** a Department of Chemistry, General Campus, Shahid Beheshti University, Tehran 1983963113, Iran; b Department of Chemistry, University of Malaya, 50603 Kuala Lumpur, Malaysia

## Abstract

The Sn^IV^ atom in each of the two independent mol­ecules in the asymmetric unit of the title compound, [Sn(CH_3_)(C_6_H_5_)(C_10_H_6_NO_2_)_2_]·H_2_O, is *N*,*O*-chelated by two quinoline-2-carboxyl­ate ions; the dative Sn—N bonds are significantly longer than the covalent Sn—O bonds. The two O and two N atoms comprise a trapezoid, and the diorganotin skeleton is bent over the longer N—N edge [C—Sn—C = 144.2 (1) and 144.5 (1)° in the two independent mol­ecules]. The uncoordinated water mol­ecules serve to connect the skew-trapezoidal bipyramidal tin-bearing mol­ecules, generating a linear chain motif running along the *ac* diagonal. The crystal studied was a non-merohedral twin having a minor component of 33.2 (1)%.

## Related literature

For other diorganotin bis­(quinoline-2-carboxyl­ates), see: Chen *et al.* (2006 ▶); Dakternieks *et al.* (2003*a* ▶,*b* ▶); Kuang *et al.* (2008*a* ▶,*b* ▶); Wang *et al.* (2004 ▶); Yin *et al.* (2005 ▶); Zhang *et al.* (2006 ▶).
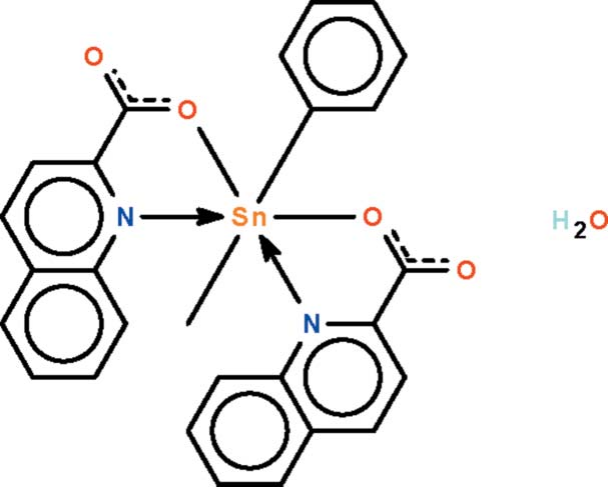


## Experimental

### 

#### Crystal data

[Sn(CH_3_)(C_6_H_5_)(C_10_H_6_NO_2_)_2_]·H_2_O
*M* *_r_* = 573.16
Triclinic, 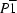

*a* = 10.1645 (5) Å
*b* = 13.9747 (6) Å
*c* = 17.0047 (8) Å
α = 103.8670 (6)°
β = 95.3560 (7)°
γ = 99.0625 (7)°
*V* = 2294.0 (2) Å^3^
*Z* = 4
Mo *K*α radiation
μ = 1.16 mm^−1^
*T* = 100 K
0.15 × 0.10 × 0.05 mm

#### Data collection

Bruker SMART APEX diffractometer
Absorption correction: multi-scan (*TWINABS*; Bruker, 2009 ▶) *T*_min_ = 0.846, *T*_max_ = 0.944
13450 measured reflections
11013 independent reflections
8760 reflections with *I* > 2σ(*I*)
*R*_int_ = 0.028

#### Refinement

*R*[*F* ^2^ > 2σ(*F* ^2^)] = 0.032
*wR*(*F* ^2^) = 0.101
*S* = 1.07
11013 reflections
634 parameters
H-atom parameters constrained
Δρ_max_ = 1.26 e Å^−3^
Δρ_min_ = −0.75 e Å^−3^

### 

Data collection: *APEX2* (Bruker, 2009 ▶); cell refinement: *SAINT* (Bruker, 2009 ▶); data reduction: *SAINT*; program(s) used to solve structure: *SHELXS97* (Sheldrick, 2008 ▶); program(s) used to refine structure: *SHELXL97* (Sheldrick, 2008 ▶); molecular graphics: *X-SEED* (Barbour, 2001 ▶); software used to prepare material for publication: *publCIF* (Westrip, 2010 ▶).

## Supplementary Material

Crystal structure: contains datablocks global, I. DOI: 10.1107/S1600536810054437/hg2770sup1.cif

Structure factors: contains datablocks I. DOI: 10.1107/S1600536810054437/hg2770Isup2.hkl

Additional supplementary materials:  crystallographic information; 3D view; checkCIF report

## Figures and Tables

**Table:** 

Sn1—O1	2.074 (2)
Sn1—C1	2.093 (3)
Sn1—O3	2.097 (2)
Sn1—C2	2.121 (3)
Sn1—N1	2.483 (3)
Sn1—N2	2.644 (3)
Sn2—O5	2.072 (2)
Sn2—O7	2.091 (2)
Sn2—C28	2.096 (3)
Sn2—C29	2.123 (3)
Sn2—N3	2.548 (3)
Sn2—N4	2.647 (3)

**Table:** 

C1—Sn1—C2	144.5 (1)
C28—Sn2—C29	144.2 (1)

**Table 2:** Hydrogen-bond geometry (Å, °)

*D*—H⋯*A*	*D*—H	H⋯*A*	*D*⋯*A*	*D*—H⋯*A*
O1w—H1w1⋯O2	0.84	2.09	2.914 (4)	167
O1w—H1w2⋯O8	0.84	1.95	2.777 (4)	167
O2w—H2w1⋯O4	0.84	2.08	2.911 (4)	171
O2w—H2w2⋯O6^i^	0.84	2.07	2.898 (4)	171

Symmetry code: (i) 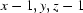.

**Fig. 1.**
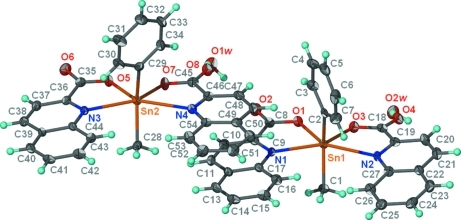
Thermal ellipsoid plot (Barbour, 2001) of the two independent formula units of Sn(CH3)(C6H5)(C10H6NO2)2.H2O at the 70% probability level. Hydrogen atoms are drawn as spheres of arbitrary radius.

**Fig. 2.**
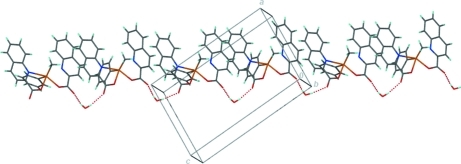
Hydrogen-bonded chain structure.

## supplementary crystallographic information

### Comment

The anion of quinoline-2-carboxylic acid *N*,*O*-chelates to diorganotin(IV) cations to confer a six-coordinate geometry to the tin atom. The geometry is better described as a skew-trapezoidal bipyramid. The reported compounds having two identical organic radicals bound to tin (see: Chen *et al.*, 2006; Dakternieks *et al.*, 2003*a*, 2003*b*; Kuang *et al.*, 2008*a*, 2008*b*; Wang *et al.*, 2004; Yin *et al.*, 2005; Zhang *et al.*, 2006). The mixed-organyl compound, Sn(CH_3_)(C_6_H_5_)(C_10_H_6_NO_2_)_2_^.^H_2_O (Scheme I) is an example of a diorganotin quinoline-2-carboxylate having different organic groups; the synthesis of the parent diorganotin dichloride is a non-trivial synthesis. There are two independent molecules in the asymmetric unit. In both, the tin atom is *N*,*O*-chelated by two carboxylate ions; the dative Sn–N bond is significantly longer than the covalent Sn–O bond. The two O and two N atoms comprise a trapezoid, and the diorganotin skeleton is bent over the longer N–N edge (Table 1, (Fig. 1). The lattice water molecules serve to connect the skew-trapezoidal bipyramidal tin-bearing molecules to generate a linear chain motif (Fig. 2).

### Experimental

Sodium quinoline-2-carboxylate was prepared by reacting sodium hydroxide and quinoline-2-carboxylic acid in toluene and removing of water in a Dean-Stark trap. The compound (0.20 g, 1 mmol) and methylphenyltin dichloride (0.35 g, 1 mmol) were stirred in a small volume of toluene at room temperature for an hour. The solid that formed was collected and recrystalized from methanol.

### Refinement

Hydrogen atoms were placed in calculated positions (C–H 0.95–0.98 Å) and included in the refinement in the riding model approximation, with *U*(H) set to 1.2–1.5*U*_eq_(C). The water H-atoms were also placed in calculated positions on the basis of hydrogen bonding interactions, with O–H set at 0.84 Å; their temperature factors were tied by a factor of 1.5 times. The final difference Fourier map had a peak in the vicinity of Sn2.

The crystal studied is a non-merohedral twin. The two twin domains were found by using *CELL_NOW*. The frames were integrated simultaneously by using *TWINABS* (Bruker, 2009). The minor component refined to 33.2 (1)%.

### Crystal data

**Table:**

[Sn(CH_3_)(C_6_H_5_)(C_10_H_6_NO_2_)_2_]·H_2_O	*Z* = 4
*M**_r_* = 573.16	*F*(000) = 1152
Triclinic, *P*1	*D*_x_ = 1.660 Mg m^−^^3^
Hall symbol: -P 1	Mo *K*α radiation, λ = 0.71073 Å
*a* = 10.1645 (5) Å	Cell parameters from 3873 reflections
*b* = 13.9747 (6) Å	θ = 2.3–28.3°
*c* = 17.0047 (8) Å	µ = 1.16 mm^−^^1^
α = 103.8670 (6)°	*T* = 100 K
β = 95.3560 (7)°	Prism, colorless
γ = 99.0625 (7)°	0.15 × 0.10 × 0.05 mm
*V* = 2294.0 (2) Å^3^	

### Data collection

**Table:**

Bruker SMART APEX diffractometer	11013 independent reflections
Radiation source: fine-focus sealed tube	8760 reflections with *I* > 2σ(*I*)
graphite	*R*_int_ = 0.028
ω scans	θ_max_ = 27.5°, θ_min_ = 1.3°
Absorption correction: multi-scan (TWINABS; Bruker, 2009)	*h* = −13→12
*T*_min_ = 0.846, *T*_max_ = 0.944	*k* = −18→17
13450 measured reflections	*l* = 0→22

### Refinement

**Table:**

Refinement on *F*^2^	Primary atom site location: structure-invariant direct methods
Least-squares matrix: full	Secondary atom site location: difference Fourier map
*R*[*F*^2^ > 2σ(*F*^2^)] = 0.032	Hydrogen site location: inferred from neighbouring sites
*wR*(*F*^2^) = 0.101	H-atom parameters constrained
*S* = 1.07	*w* = 1/[σ^2^(*F*_o_^2^) + (0.0569*P*)^2^] where *P* = (*F*_o_^2^ + 2*F*_c_^2^)/3
11013 reflections	(Δ/σ)_max_ = 0.001
634 parameters	Δρ_max_ = 1.26 e Å^−^^3^
0 restraints	Δρ_min_ = −0.75 e Å^−^^3^

### Fractional atomic coordinates and isotropic or equivalent isotropic displacement parameters (Å^2^)

**Table:**

	*x*	*y*	*z*	*U*_iso_*/*U*_eq_	
Sn1	0.41077 (2)	0.201996 (17)	0.167572 (14)	0.01248 (7)	
Sn2	0.86475 (2)	0.207815 (17)	0.668056 (14)	0.01286 (7)	
O1	0.2924 (2)	0.15333 (18)	0.24844 (14)	0.0156 (5)	
O2	0.2587 (3)	0.0812 (2)	0.34970 (16)	0.0226 (6)	
O3	0.2166 (2)	0.21193 (19)	0.12076 (15)	0.0180 (5)	
O4	0.0655 (2)	0.24855 (19)	0.03525 (15)	0.0198 (6)	
O5	0.7572 (2)	0.14924 (18)	0.74893 (14)	0.0160 (5)	
O6	0.7364 (3)	0.0812 (2)	0.85284 (15)	0.0222 (6)	
O7	0.6652 (2)	0.20711 (19)	0.62455 (15)	0.0172 (5)	
O8	0.5004 (2)	0.24978 (19)	0.55227 (15)	0.0199 (5)	
O1W	0.2494 (3)	0.1263 (2)	0.52532 (17)	0.0306 (7)	
H1W1	0.2399	0.1075	0.4740	0.046*	
H1W2	0.3242	0.1652	0.5414	0.046*	
O2W	−0.1866 (3)	0.1123 (2)	0.02681 (16)	0.0253 (6)	
H2W1	−0.1125	0.1520	0.0349	0.038*	
H2W2	−0.2168	0.0989	−0.0231	0.038*	
N1	0.5542 (3)	0.1471 (2)	0.26831 (16)	0.0126 (6)	
N2	0.4156 (3)	0.2811 (2)	0.04111 (17)	0.0133 (6)	
N3	1.0246 (3)	0.1538 (2)	0.76648 (17)	0.0127 (6)	
N4	0.8479 (3)	0.2890 (2)	0.54278 (17)	0.0152 (6)	
C1	0.4553 (4)	0.0806 (3)	0.0820 (2)	0.0170 (7)	
H1A	0.4042	0.0737	0.0285	0.026*	
H1B	0.4307	0.0190	0.0995	0.026*	
H1C	0.5517	0.0922	0.0780	0.026*	
C2	0.4859 (3)	0.3512 (3)	0.2381 (2)	0.0142 (7)	
C3	0.4313 (4)	0.3852 (3)	0.3102 (2)	0.0190 (8)	
H3	0.3638	0.3414	0.3262	0.023*	
C4	0.4761 (4)	0.4835 (3)	0.3588 (2)	0.0207 (8)	
H4	0.4381	0.5061	0.4074	0.025*	
C5	0.5747 (4)	0.5474 (3)	0.3366 (2)	0.0226 (8)	
H5	0.6022	0.6147	0.3686	0.027*	
C6	0.6333 (4)	0.5135 (3)	0.2678 (2)	0.0247 (9)	
H6	0.7050	0.5567	0.2546	0.030*	
C7	0.5895 (4)	0.4180 (3)	0.2180 (2)	0.0219 (8)	
H7	0.6291	0.3967	0.1699	0.026*	
C8	0.3337 (3)	0.1119 (3)	0.3054 (2)	0.0146 (7)	
C9	0.4796 (3)	0.1039 (2)	0.3154 (2)	0.0141 (7)	
C10	0.5301 (4)	0.0553 (3)	0.3709 (2)	0.0179 (7)	
H10	0.4718	0.0247	0.4020	0.021*	
C11	0.6637 (4)	0.0521 (3)	0.3800 (2)	0.0174 (7)	
H11	0.6994	0.0179	0.4166	0.021*	
C12	0.7490 (3)	0.1006 (3)	0.3340 (2)	0.0149 (7)	
C13	0.8910 (4)	0.1051 (3)	0.3419 (2)	0.0190 (8)	
H13	0.9328	0.0773	0.3813	0.023*	
C14	0.9666 (4)	0.1483 (3)	0.2940 (2)	0.0202 (8)	
H14	1.0606	0.1489	0.2988	0.024*	
C15	0.9072 (4)	0.1927 (3)	0.2371 (2)	0.0203 (8)	
H15	0.9620	0.2229	0.2041	0.024*	
C16	0.7725 (4)	0.1931 (3)	0.2284 (2)	0.0180 (7)	
H16	0.7340	0.2239	0.1901	0.022*	
C17	0.6905 (3)	0.1472 (2)	0.2772 (2)	0.0143 (7)	
C18	0.1825 (4)	0.2494 (3)	0.0607 (2)	0.0158 (7)	
C19	0.2928 (3)	0.2959 (3)	0.0211 (2)	0.0155 (7)	
C20	0.2613 (4)	0.3494 (3)	−0.0358 (2)	0.0189 (8)	
H20	0.1719	0.3595	−0.0471	0.023*	
C21	0.3605 (4)	0.3867 (3)	−0.0745 (2)	0.0212 (8)	
H21	0.3412	0.4244	−0.1124	0.025*	
C22	0.4925 (4)	0.3695 (3)	−0.0584 (2)	0.0166 (7)	
C23	0.6027 (4)	0.4037 (3)	−0.0975 (2)	0.0217 (8)	
H23	0.5897	0.4434	−0.1347	0.026*	
C24	0.7252 (4)	0.3804 (3)	−0.0822 (2)	0.0216 (8)	
H24	0.7969	0.4026	−0.1095	0.026*	
C25	0.7464 (4)	0.3223 (3)	−0.0252 (2)	0.0206 (8)	
H25	0.8326	0.3062	−0.0147	0.025*	
C26	0.6448 (4)	0.2897 (3)	0.0145 (2)	0.0190 (8)	
H26	0.6603	0.2508	0.0520	0.023*	
C27	0.5161 (4)	0.3138 (2)	−0.0002 (2)	0.0152 (7)	
C28	0.9242 (4)	0.0934 (2)	0.5824 (2)	0.0152 (7)	
H28A	0.8668	0.0798	0.5299	0.023*	
H28B	0.9157	0.0325	0.6019	0.023*	
H28C	1.0181	0.1145	0.5754	0.023*	
C29	0.9229 (4)	0.3589 (3)	0.7386 (2)	0.0145 (7)	
C30	1.0373 (4)	0.3907 (3)	0.7967 (2)	0.0206 (8)	
H30	1.0942	0.3445	0.8033	0.025*	
C31	1.0698 (4)	0.4887 (3)	0.8452 (2)	0.0237 (9)	
H31	1.1490	0.5093	0.8840	0.028*	
C32	0.9867 (4)	0.5561 (3)	0.8369 (2)	0.0219 (8)	
H32	1.0087	0.6231	0.8701	0.026*	
C33	0.8723 (4)	0.5264 (3)	0.7808 (2)	0.0205 (8)	
H33	0.8149	0.5726	0.7755	0.025*	
C34	0.8404 (4)	0.4280 (3)	0.7314 (2)	0.0189 (8)	
H34	0.7615	0.4080	0.6924	0.023*	
C35	0.8066 (4)	0.1124 (3)	0.8058 (2)	0.0158 (7)	
C36	0.9541 (3)	0.1074 (2)	0.8130 (2)	0.0130 (7)	
C37	1.0090 (4)	0.0569 (3)	0.8658 (2)	0.0158 (7)	
H37	0.9527	0.0230	0.8961	0.019*	
C38	1.1431 (4)	0.0562 (3)	0.8737 (2)	0.0160 (7)	
H38	1.1821	0.0227	0.9097	0.019*	
C39	1.2237 (4)	0.1072 (3)	0.8266 (2)	0.0144 (7)	
C40	1.3655 (4)	0.1122 (3)	0.8324 (2)	0.0172 (7)	
H40	1.4097	0.0838	0.8704	0.021*	
C41	1.4375 (3)	0.1576 (3)	0.7837 (2)	0.0175 (7)	
H41	1.5314	0.1582	0.7862	0.021*	
C42	1.3746 (3)	0.2036 (3)	0.7296 (2)	0.0183 (8)	
H42	1.4271	0.2362	0.6969	0.022*	
C43	1.2386 (3)	0.2024 (3)	0.7232 (2)	0.0148 (7)	
H43	1.1973	0.2334	0.6860	0.018*	
C44	1.1604 (3)	0.1547 (2)	0.7724 (2)	0.0126 (7)	
C45	0.6194 (4)	0.2467 (3)	0.5688 (2)	0.0169 (7)	
C46	0.7199 (3)	0.2916 (3)	0.5223 (2)	0.0154 (7)	
C47	0.6736 (4)	0.3338 (3)	0.4599 (2)	0.0176 (7)	
H47	0.5805	0.3335	0.4476	0.021*	
C48	0.7653 (4)	0.3754 (3)	0.4173 (2)	0.0183 (8)	
H48	0.7367	0.4054	0.3758	0.022*	
C49	0.9024 (4)	0.3727 (3)	0.4362 (2)	0.0167 (7)	
C50	1.0024 (4)	0.4138 (3)	0.3954 (2)	0.0205 (8)	
H50	0.9780	0.4460	0.3544	0.025*	
C51	1.1345 (4)	0.4080 (3)	0.4141 (2)	0.0210 (8)	
H51	1.2011	0.4353	0.3858	0.025*	
C52	1.1709 (4)	0.3611 (3)	0.4756 (2)	0.0213 (8)	
H52	1.2624	0.3568	0.4882	0.026*	
C53	1.0764 (4)	0.3216 (3)	0.5173 (2)	0.0215 (8)	
H53	1.1024	0.2898	0.5582	0.026*	
C54	0.9403 (3)	0.3284 (3)	0.4993 (2)	0.0142 (7)	

### Atomic displacement parameters (Å^2^)

**Table:**

	*U*^11^	*U*^22^	*U*^33^	*U*^12^	*U*^13^	*U*^23^
Sn1	0.00909 (12)	0.01302 (12)	0.01558 (13)	0.00170 (9)	0.00211 (9)	0.00426 (9)
Sn2	0.00969 (13)	0.01338 (12)	0.01618 (13)	0.00100 (9)	0.00245 (9)	0.00557 (9)
O1	0.0116 (12)	0.0182 (12)	0.0189 (12)	0.0018 (10)	0.0035 (10)	0.0082 (10)
O2	0.0175 (14)	0.0297 (15)	0.0245 (14)	0.0029 (11)	0.0085 (11)	0.0132 (12)
O3	0.0123 (13)	0.0244 (14)	0.0189 (13)	0.0035 (10)	0.0020 (10)	0.0086 (11)
O4	0.0134 (13)	0.0215 (13)	0.0235 (14)	0.0044 (10)	−0.0034 (10)	0.0053 (11)
O5	0.0089 (12)	0.0236 (13)	0.0185 (13)	0.0023 (10)	0.0024 (10)	0.0112 (10)
O6	0.0200 (14)	0.0267 (14)	0.0221 (14)	0.0018 (11)	0.0077 (11)	0.0106 (11)
O7	0.0096 (12)	0.0241 (13)	0.0209 (13)	0.0011 (10)	0.0006 (10)	0.0135 (11)
O8	0.0121 (13)	0.0254 (14)	0.0239 (14)	0.0030 (11)	0.0016 (10)	0.0103 (11)
O1W	0.0236 (16)	0.0369 (17)	0.0289 (16)	−0.0037 (13)	0.0011 (12)	0.0106 (13)
O2W	0.0203 (14)	0.0314 (15)	0.0254 (14)	0.0016 (12)	0.0041 (11)	0.0113 (12)
N1	0.0131 (15)	0.0151 (14)	0.0113 (14)	0.0041 (11)	0.0022 (11)	0.0057 (11)
N2	0.0154 (15)	0.0136 (14)	0.0124 (14)	0.0025 (11)	0.0014 (11)	0.0068 (11)
N3	0.0117 (15)	0.0148 (14)	0.0131 (14)	0.0039 (11)	0.0021 (11)	0.0049 (11)
N4	0.0141 (15)	0.0157 (15)	0.0159 (15)	0.0029 (12)	0.0012 (11)	0.0045 (12)
C1	0.0173 (19)	0.0160 (17)	0.0175 (17)	0.0041 (14)	0.0028 (14)	0.0030 (14)
C2	0.0137 (18)	0.0158 (17)	0.0154 (17)	0.0067 (14)	−0.0001 (13)	0.0069 (14)
C3	0.0107 (18)	0.0181 (18)	0.027 (2)	0.0018 (14)	0.0020 (14)	0.0046 (15)
C4	0.0125 (18)	0.0225 (19)	0.024 (2)	0.0071 (15)	−0.0011 (15)	0.0000 (16)
C5	0.026 (2)	0.0130 (17)	0.026 (2)	0.0051 (15)	−0.0056 (16)	0.0027 (15)
C6	0.029 (2)	0.0179 (19)	0.026 (2)	−0.0044 (16)	−0.0012 (17)	0.0120 (16)
C7	0.024 (2)	0.0187 (19)	0.0208 (19)	0.0007 (16)	0.0026 (15)	0.0031 (15)
C8	0.0112 (17)	0.0146 (17)	0.0176 (17)	0.0009 (13)	0.0011 (13)	0.0044 (14)
C9	0.0139 (18)	0.0114 (16)	0.0156 (17)	0.0022 (13)	−0.0002 (13)	0.0016 (13)
C10	0.0195 (19)	0.0150 (17)	0.0187 (18)	0.0011 (14)	0.0021 (14)	0.0050 (14)
C11	0.024 (2)	0.0150 (17)	0.0131 (17)	0.0033 (15)	−0.0014 (14)	0.0044 (14)
C12	0.0133 (18)	0.0159 (17)	0.0149 (17)	0.0058 (14)	−0.0013 (13)	0.0023 (14)
C13	0.0134 (18)	0.0213 (19)	0.0200 (18)	0.0076 (15)	−0.0061 (14)	0.0012 (15)
C14	0.0126 (18)	0.0190 (18)	0.026 (2)	0.0039 (14)	0.0013 (15)	−0.0007 (15)
C15	0.0154 (19)	0.0178 (18)	0.026 (2)	0.0004 (15)	0.0046 (15)	0.0028 (15)
C16	0.0171 (19)	0.0155 (17)	0.0206 (18)	0.0021 (14)	0.0015 (14)	0.0042 (14)
C17	0.0139 (18)	0.0137 (17)	0.0146 (17)	0.0035 (14)	−0.0006 (13)	0.0025 (13)
C18	0.0145 (18)	0.0128 (16)	0.0190 (18)	0.0034 (14)	0.0023 (14)	0.0013 (14)
C19	0.0133 (18)	0.0143 (17)	0.0171 (17)	0.0022 (14)	0.0011 (14)	0.0013 (14)
C20	0.0139 (18)	0.0207 (19)	0.0245 (19)	0.0045 (15)	0.0005 (15)	0.0102 (16)
C21	0.025 (2)	0.0185 (19)	0.0203 (19)	0.0034 (16)	−0.0017 (15)	0.0068 (15)
C22	0.0172 (19)	0.0155 (17)	0.0172 (17)	0.0045 (14)	0.0003 (14)	0.0042 (14)
C23	0.029 (2)	0.0187 (19)	0.0190 (19)	0.0023 (16)	0.0028 (16)	0.0094 (15)
C24	0.025 (2)	0.0207 (19)	0.0180 (18)	−0.0011 (16)	0.0078 (15)	0.0049 (15)
C25	0.0184 (19)	0.025 (2)	0.0191 (18)	0.0051 (16)	0.0042 (15)	0.0056 (15)
C26	0.0168 (19)	0.0162 (18)	0.0231 (19)	0.0002 (14)	0.0010 (15)	0.0057 (15)
C27	0.0159 (18)	0.0107 (16)	0.0166 (17)	−0.0023 (13)	0.0002 (14)	0.0027 (13)
C28	0.0176 (18)	0.0155 (17)	0.0132 (16)	0.0014 (14)	0.0042 (13)	0.0054 (14)
C29	0.0161 (18)	0.0147 (17)	0.0147 (16)	0.0032 (14)	0.0069 (13)	0.0056 (14)
C30	0.0176 (19)	0.0175 (18)	0.027 (2)	0.0066 (15)	0.0016 (15)	0.0044 (15)
C31	0.018 (2)	0.0145 (18)	0.034 (2)	0.0012 (15)	−0.0005 (16)	0.0008 (16)
C32	0.026 (2)	0.0114 (17)	0.027 (2)	0.0027 (15)	0.0066 (16)	0.0038 (15)
C33	0.024 (2)	0.0170 (18)	0.026 (2)	0.0102 (15)	0.0050 (16)	0.0099 (15)
C34	0.0191 (19)	0.0221 (19)	0.0182 (18)	0.0068 (15)	0.0017 (14)	0.0087 (15)
C35	0.0151 (18)	0.0151 (17)	0.0167 (17)	0.0013 (14)	0.0040 (14)	0.0037 (14)
C36	0.0163 (18)	0.0098 (15)	0.0124 (16)	−0.0005 (13)	0.0030 (13)	0.0033 (13)
C37	0.0188 (19)	0.0137 (17)	0.0152 (17)	0.0001 (14)	0.0036 (14)	0.0055 (14)
C38	0.0197 (19)	0.0138 (17)	0.0141 (17)	0.0025 (14)	0.0008 (14)	0.0036 (13)
C39	0.0182 (18)	0.0133 (16)	0.0127 (16)	0.0062 (14)	0.0013 (13)	0.0038 (13)
C40	0.0176 (19)	0.0152 (17)	0.0180 (18)	0.0054 (14)	−0.0020 (14)	0.0030 (14)
C41	0.0069 (17)	0.0186 (18)	0.0240 (19)	0.0034 (14)	0.0009 (14)	−0.0003 (15)
C42	0.0075 (17)	0.0211 (18)	0.0236 (19)	−0.0004 (14)	0.0029 (14)	0.0023 (15)
C43	0.0124 (18)	0.0157 (17)	0.0161 (17)	0.0007 (13)	0.0028 (13)	0.0049 (14)
C44	0.0110 (17)	0.0125 (16)	0.0134 (16)	0.0035 (13)	−0.0010 (13)	0.0019 (13)
C45	0.0156 (19)	0.0138 (17)	0.0185 (18)	0.0000 (14)	0.0015 (14)	0.0008 (14)
C46	0.0130 (18)	0.0146 (17)	0.0176 (17)	0.0018 (14)	0.0014 (14)	0.0031 (14)
C47	0.0130 (18)	0.0190 (18)	0.0206 (18)	0.0057 (14)	0.0014 (14)	0.0029 (15)
C48	0.022 (2)	0.0160 (17)	0.0181 (18)	0.0049 (15)	0.0005 (15)	0.0071 (14)
C49	0.0187 (19)	0.0120 (16)	0.0185 (18)	0.0017 (14)	0.0044 (14)	0.0020 (14)
C50	0.019 (2)	0.0185 (18)	0.025 (2)	0.0031 (15)	0.0030 (15)	0.0081 (16)
C51	0.022 (2)	0.0183 (18)	0.0218 (19)	−0.0014 (15)	0.0044 (15)	0.0065 (15)
C52	0.0130 (19)	0.029 (2)	0.0215 (19)	0.0021 (15)	0.0029 (15)	0.0070 (16)
C53	0.0127 (19)	0.026 (2)	0.026 (2)	0.0035 (15)	−0.0023 (15)	0.0074 (16)
C54	0.0108 (17)	0.0133 (16)	0.0173 (17)	0.0009 (13)	0.0032 (13)	0.0021 (13)

### Geometric parameters (Å, °)

**Table:**

Sn1—O1	2.074 (2)	C18—C19	1.502 (5)
Sn1—C1	2.093 (3)	C19—C20	1.400 (5)
Sn1—O3	2.097 (2)	C20—C21	1.359 (5)
Sn1—C2	2.121 (3)	C20—H20	0.9500
Sn1—N1	2.483 (3)	C21—C22	1.413 (5)
Sn1—N2	2.644 (3)	C21—H21	0.9500
Sn2—O5	2.072 (2)	C22—C27	1.424 (5)
Sn2—O7	2.091 (2)	C22—C23	1.431 (5)
Sn2—C28	2.096 (3)	C23—C24	1.353 (5)
Sn2—C29	2.123 (3)	C23—H23	0.9500
Sn2—N3	2.548 (3)	C24—C25	1.429 (5)
Sn2—N4	2.647 (3)	C24—H24	0.9500
O1—C8	1.311 (4)	C25—C26	1.364 (5)
O2—C8	1.222 (4)	C25—H25	0.9500
O3—C18	1.298 (4)	C26—C27	1.415 (5)
O4—C18	1.224 (4)	C26—H26	0.9500
O5—C35	1.297 (4)	C28—H28A	0.9800
O6—C35	1.231 (4)	C28—H28B	0.9800
O7—C45	1.293 (4)	C28—H28C	0.9800
O8—C45	1.226 (4)	C29—C30	1.392 (5)
O1W—H1W1	0.8401	C29—C34	1.394 (5)
O1W—H1W2	0.8407	C30—C31	1.389 (5)
O2W—H2W1	0.8407	C30—H30	0.9500
O2W—H2W2	0.8406	C31—C32	1.384 (5)
N1—C9	1.334 (4)	C31—H31	0.9500
N1—C17	1.379 (4)	C32—C33	1.373 (5)
N2—C19	1.324 (4)	C32—H32	0.9500
N2—C27	1.374 (4)	C33—C34	1.399 (5)
N3—C36	1.328 (4)	C33—H33	0.9500
N3—C44	1.373 (4)	C34—H34	0.9500
N4—C46	1.323 (4)	C35—C36	1.506 (5)
N4—C54	1.376 (4)	C36—C37	1.398 (5)
C1—H1A	0.9800	C37—C38	1.359 (5)
C1—H1B	0.9800	C37—H37	0.9500
C1—H1C	0.9800	C38—C39	1.425 (5)
C2—C3	1.402 (5)	C38—H38	0.9500
C2—C7	1.417 (5)	C39—C44	1.423 (5)
C3—C4	1.402 (5)	C39—C40	1.424 (5)
C3—H3	0.9500	C40—C41	1.358 (5)
C4—C5	1.377 (5)	C40—H40	0.9500
C4—H4	0.9500	C41—C42	1.404 (5)
C5—C6	1.379 (5)	C41—H41	0.9500
C5—H5	0.9500	C42—C43	1.374 (5)
C6—C7	1.378 (5)	C42—H42	0.9500
C6—H6	0.9500	C43—C44	1.414 (5)
C7—H7	0.9500	C43—H43	0.9500
C8—C9	1.502 (5)	C45—C46	1.499 (5)
C9—C10	1.393 (5)	C46—C47	1.410 (5)
C10—C11	1.362 (5)	C47—C48	1.374 (5)
C10—H10	0.9500	C47—H47	0.9500
C11—C12	1.422 (5)	C48—C49	1.409 (5)
C11—H11	0.9500	C48—H48	0.9500
C12—C17	1.424 (5)	C49—C50	1.409 (5)
C12—C13	1.427 (5)	C49—C54	1.413 (5)
C13—C14	1.351 (5)	C50—C51	1.370 (5)
C13—H13	0.9500	C50—H50	0.9500
C14—C15	1.407 (5)	C51—C52	1.412 (5)
C14—H14	0.9500	C51—H51	0.9500
C15—C16	1.365 (5)	C52—C53	1.370 (5)
C15—H15	0.9500	C52—H52	0.9500
C16—C17	1.417 (5)	C53—C54	1.412 (5)
C16—H16	0.9500	C53—H53	0.9500
			
O1—Sn1—C1	111.11 (12)	C21—C20—C19	119.0 (3)
O1—Sn1—O3	76.84 (9)	C21—C20—H20	120.5
C1—Sn1—O3	103.41 (12)	C19—C20—H20	120.5
O1—Sn1—C2	98.68 (11)	C20—C21—C22	120.0 (3)
C1—Sn1—C2	144.5 (1)	C20—C21—H21	120.0
O3—Sn1—C2	101.75 (11)	C22—C21—H21	120.0
O1—Sn1—N1	71.06 (9)	C21—C22—C27	117.6 (3)
C1—Sn1—N1	84.82 (11)	C21—C22—C23	124.0 (3)
O3—Sn1—N1	147.65 (9)	C27—C22—C23	118.4 (3)
C2—Sn1—N1	87.16 (11)	C24—C23—C22	121.1 (3)
O1—Sn1—N2	145.43 (9)	C24—C23—H23	119.5
C1—Sn1—N2	81.50 (11)	C22—C23—H23	119.5
O3—Sn1—N2	68.83 (9)	C23—C24—C25	119.9 (3)
C2—Sn1—N2	84.72 (11)	C23—C24—H24	120.1
N1—Sn1—N2	143.44 (9)	C25—C24—H24	120.1
O5—Sn2—O7	76.83 (9)	C26—C25—C24	121.0 (3)
O5—Sn2—C28	110.72 (12)	C26—C25—H25	119.5
O7—Sn2—C28	105.40 (12)	C24—C25—H25	119.5
O5—Sn2—C29	99.13 (11)	C25—C26—C27	120.1 (3)
O7—Sn2—C29	100.21 (12)	C25—C26—H26	120.0
C28—Sn2—C29	144.2 (1)	C27—C26—H26	120.0
O5—Sn2—N3	70.49 (9)	N2—C27—C26	119.1 (3)
O7—Sn2—N3	146.93 (9)	N2—C27—C22	121.3 (3)
C28—Sn2—N3	82.07 (11)	C26—C27—C22	119.6 (3)
C29—Sn2—N3	89.99 (11)	Sn2—C28—H28A	109.5
O5—Sn2—N4	144.85 (9)	Sn2—C28—H28B	109.5
O7—Sn2—N4	68.19 (9)	H28A—C28—H28B	109.5
C28—Sn2—N4	82.64 (11)	Sn2—C28—H28C	109.5
C29—Sn2—N4	84.11 (11)	H28A—C28—H28C	109.5
N3—Sn2—N4	144.65 (9)	H28B—C28—H28C	109.5
C8—O1—Sn1	125.0 (2)	C30—C29—C34	118.0 (3)
C18—O3—Sn1	127.6 (2)	C30—C29—Sn2	122.1 (3)
C35—O5—Sn2	125.8 (2)	C34—C29—Sn2	119.8 (3)
C45—O7—Sn2	129.0 (2)	C31—C30—C29	121.2 (3)
H1W1—O1W—H1W2	108.1	C31—C30—H30	119.4
H2W1—O2W—H2W2	108.2	C29—C30—H30	119.4
C9—N1—C17	118.2 (3)	C32—C31—C30	119.9 (4)
C9—N1—Sn1	110.9 (2)	C32—C31—H31	120.1
C17—N1—Sn1	130.6 (2)	C30—C31—H31	120.1
C19—N2—C27	118.3 (3)	C33—C32—C31	120.1 (3)
C19—N2—Sn1	107.7 (2)	C33—C32—H32	120.0
C27—N2—Sn1	133.8 (2)	C31—C32—H32	120.0
C36—N3—C44	118.1 (3)	C32—C33—C34	120.0 (3)
C36—N3—Sn2	109.4 (2)	C32—C33—H33	120.0
C44—N3—Sn2	132.1 (2)	C34—C33—H33	120.0
C46—N4—C54	117.6 (3)	C29—C34—C33	120.8 (3)
C46—N4—Sn2	108.1 (2)	C29—C34—H34	119.6
C54—N4—Sn2	134.3 (2)	C33—C34—H34	119.6
Sn1—C1—H1A	109.5	O6—C35—O5	121.9 (3)
Sn1—C1—H1B	109.5	O6—C35—C36	120.0 (3)
H1A—C1—H1B	109.5	O5—C35—C36	118.2 (3)
Sn1—C1—H1C	109.5	N3—C36—C37	124.0 (3)
H1A—C1—H1C	109.5	N3—C36—C35	115.4 (3)
H1B—C1—H1C	109.5	C37—C36—C35	120.6 (3)
C3—C2—C7	117.9 (3)	C38—C37—C36	119.8 (3)
C3—C2—Sn1	117.8 (3)	C38—C37—H37	120.1
C7—C2—Sn1	124.2 (2)	C36—C37—H37	120.1
C4—C3—C2	120.3 (4)	C37—C38—C39	118.3 (3)
C4—C3—H3	119.8	C37—C38—H38	120.8
C2—C3—H3	119.8	C39—C38—H38	120.8
C5—C4—C3	120.4 (3)	C44—C39—C40	119.1 (3)
C5—C4—H4	119.8	C44—C39—C38	118.8 (3)
C3—C4—H4	119.8	C40—C39—C38	122.1 (3)
C4—C5—C6	119.9 (3)	C41—C40—C39	120.1 (3)
C4—C5—H5	120.1	C41—C40—H40	120.0
C6—C5—H5	120.1	C39—C40—H40	120.0
C7—C6—C5	121.0 (4)	C40—C41—C42	120.7 (3)
C7—C6—H6	119.5	C40—C41—H41	119.7
C5—C6—H6	119.5	C42—C41—H41	119.7
C6—C7—C2	120.5 (3)	C43—C42—C41	121.2 (3)
C6—C7—H7	119.8	C43—C42—H42	119.4
C2—C7—H7	119.8	C41—C42—H42	119.4
O2—C8—O1	122.5 (3)	C42—C43—C44	119.5 (3)
O2—C8—C9	120.2 (3)	C42—C43—H43	120.2
O1—C8—C9	117.3 (3)	C44—C43—H43	120.2
N1—C9—C10	124.0 (3)	N3—C44—C43	119.7 (3)
N1—C9—C8	115.0 (3)	N3—C44—C39	120.9 (3)
C10—C9—C8	121.0 (3)	C43—C44—C39	119.4 (3)
C11—C10—C9	119.4 (3)	O8—C45—O7	124.1 (3)
C11—C10—H10	120.3	O8—C45—C46	118.8 (3)
C9—C10—H10	120.3	O7—C45—C46	117.1 (3)
C10—C11—C12	119.0 (3)	N4—C46—C47	123.9 (3)
C10—C11—H11	120.5	N4—C46—C45	117.4 (3)
C12—C11—H11	120.5	C47—C46—C45	118.8 (3)
C11—C12—C17	118.6 (3)	C48—C47—C46	119.0 (3)
C11—C12—C13	123.2 (3)	C48—C47—H47	120.5
C17—C12—C13	118.2 (3)	C46—C47—H47	120.5
C14—C13—C12	120.7 (3)	C47—C48—C49	118.9 (3)
C14—C13—H13	119.6	C47—C48—H48	120.5
C12—C13—H13	119.6	C49—C48—H48	120.5
C13—C14—C15	120.5 (3)	C50—C49—C48	122.3 (3)
C13—C14—H14	119.8	C50—C49—C54	119.2 (3)
C15—C14—H14	119.8	C48—C49—C54	118.5 (3)
C16—C15—C14	121.4 (4)	C51—C50—C49	120.8 (4)
C16—C15—H15	119.3	C51—C50—H50	119.6
C14—C15—H15	119.3	C49—C50—H50	119.6
C15—C16—C17	119.3 (3)	C50—C51—C52	119.6 (3)
C15—C16—H16	120.3	C50—C51—H51	120.2
C17—C16—H16	120.3	C52—C51—H51	120.2
N1—C17—C16	119.5 (3)	C53—C52—C51	121.1 (3)
N1—C17—C12	120.6 (3)	C53—C52—H52	119.4
C16—C17—C12	119.8 (3)	C51—C52—H52	119.4
O4—C18—O3	123.2 (3)	C52—C53—C54	119.8 (4)
O4—C18—C19	118.9 (3)	C52—C53—H53	120.1
O3—C18—C19	117.9 (3)	C54—C53—H53	120.1
N2—C19—C20	123.7 (3)	N4—C54—C53	118.5 (3)
N2—C19—C18	116.9 (3)	N4—C54—C49	122.1 (3)
C20—C19—C18	119.4 (3)	C53—C54—C49	119.4 (3)
			
C1—Sn1—O1—C8	70.7 (3)	Sn1—O3—C18—O4	177.0 (2)
O3—Sn1—O1—C8	170.2 (3)	Sn1—O3—C18—C19	−3.0 (4)
C2—Sn1—O1—C8	−89.6 (3)	C27—N2—C19—C20	−4.8 (5)
N1—Sn1—O1—C8	−5.7 (2)	Sn1—N2—C19—C20	170.8 (3)
N2—Sn1—O1—C8	177.1 (2)	C27—N2—C19—C18	173.5 (3)
O1—Sn1—O3—C18	173.7 (3)	Sn1—N2—C19—C18	−11.0 (3)
C1—Sn1—O3—C18	−77.4 (3)	O4—C18—C19—N2	−169.4 (3)
C2—Sn1—O3—C18	77.4 (3)	O3—C18—C19—N2	10.6 (5)
N1—Sn1—O3—C18	−179.1 (2)	O4—C18—C19—C20	9.0 (5)
N2—Sn1—O3—C18	−2.2 (3)	O3—C18—C19—C20	−171.0 (3)
O7—Sn2—O5—C35	173.0 (3)	N2—C19—C20—C21	1.6 (5)
C28—Sn2—O5—C35	71.3 (3)	C18—C19—C20—C21	−176.6 (3)
C29—Sn2—O5—C35	−88.6 (3)	C19—C20—C21—C22	1.3 (5)
N3—Sn2—O5—C35	−1.8 (3)	C20—C21—C22—C27	−0.9 (5)
N4—Sn2—O5—C35	178.7 (2)	C20—C21—C22—C23	178.5 (4)
O5—Sn2—O7—C45	170.8 (3)	C21—C22—C23—C24	−176.7 (3)
C28—Sn2—O7—C45	−81.0 (3)	C27—C22—C23—C24	2.7 (5)
C29—Sn2—O7—C45	73.7 (3)	C22—C23—C24—C25	−1.2 (5)
N3—Sn2—O7—C45	179.8 (2)	C23—C24—C25—C26	0.0 (6)
N4—Sn2—O7—C45	−5.6 (3)	C24—C25—C26—C27	−0.4 (5)
O1—Sn1—N1—C9	7.5 (2)	C19—N2—C27—C26	−173.6 (3)
C1—Sn1—N1—C9	−106.9 (2)	Sn1—N2—C27—C26	12.2 (5)
O3—Sn1—N1—C9	0.1 (3)	C19—N2—C27—C22	5.1 (5)
C2—Sn1—N1—C9	107.7 (2)	Sn1—N2—C27—C22	−169.0 (2)
N2—Sn1—N1—C9	−175.09 (19)	C25—C26—C27—N2	−179.3 (3)
O1—Sn1—N1—C17	−178.5 (3)	C25—C26—C27—C22	2.0 (5)
C1—Sn1—N1—C17	67.1 (3)	C21—C22—C27—N2	−2.3 (5)
O3—Sn1—N1—C17	174.1 (2)	C23—C22—C27—N2	178.3 (3)
C2—Sn1—N1—C17	−78.3 (3)	C21—C22—C27—C26	176.4 (3)
N2—Sn1—N1—C17	−1.1 (4)	C23—C22—C27—C26	−3.0 (5)
O1—Sn1—N2—C19	0.1 (3)	O5—Sn2—C29—C30	85.4 (3)
C1—Sn1—N2—C19	115.3 (2)	O7—Sn2—C29—C30	163.6 (3)
O3—Sn1—N2—C19	7.2 (2)	C28—Sn2—C29—C30	−61.1 (4)
C2—Sn1—N2—C19	−97.5 (2)	N3—Sn2—C29—C30	15.2 (3)
N1—Sn1—N2—C19	−175.5 (2)	N4—Sn2—C29—C30	−129.9 (3)
O1—Sn1—N2—C27	174.7 (3)	O5—Sn2—C29—C34	−90.1 (3)
C1—Sn1—N2—C27	−70.2 (3)	O7—Sn2—C29—C34	−12.0 (3)
O3—Sn1—N2—C27	−178.2 (3)	C28—Sn2—C29—C34	123.3 (3)
C2—Sn1—N2—C27	77.0 (3)	N3—Sn2—C29—C34	−160.4 (3)
N1—Sn1—N2—C27	−1.0 (4)	N4—Sn2—C29—C34	54.6 (3)
O5—Sn2—N3—C36	6.1 (2)	C34—C29—C30—C31	−1.0 (6)
O7—Sn2—N3—C36	−3.2 (3)	Sn2—C29—C30—C31	−176.6 (3)
C28—Sn2—N3—C36	−109.3 (2)	C29—C30—C31—C32	0.9 (6)
C29—Sn2—N3—C36	105.7 (2)	C30—C31—C32—C33	−0.1 (6)
N4—Sn2—N3—C36	−174.48 (19)	C31—C32—C33—C34	−0.6 (6)
O5—Sn2—N3—C44	178.0 (3)	C30—C29—C34—C33	0.3 (5)
O7—Sn2—N3—C44	168.7 (2)	Sn2—C29—C34—C33	176.0 (3)
C28—Sn2—N3—C44	62.6 (3)	C32—C33—C34—C29	0.5 (6)
C29—Sn2—N3—C44	−82.3 (3)	Sn2—O5—C35—O6	178.2 (2)
N4—Sn2—N3—C44	−2.6 (4)	Sn2—O5—C35—C36	−2.4 (4)
O5—Sn2—N4—C46	−2.0 (3)	C44—N3—C36—C37	−2.0 (5)
O7—Sn2—N4—C46	4.0 (2)	Sn2—N3—C36—C37	171.2 (3)
C28—Sn2—N4—C46	113.8 (2)	C44—N3—C36—C35	177.9 (3)
C29—Sn2—N4—C46	−99.5 (2)	Sn2—N3—C36—C35	−8.9 (3)
N3—Sn2—N4—C46	178.9 (2)	O6—C35—C36—N3	−172.1 (3)
O5—Sn2—N4—C54	176.2 (3)	O5—C35—C36—N3	8.5 (4)
O7—Sn2—N4—C54	−177.8 (3)	O6—C35—C36—C37	7.8 (5)
C28—Sn2—N4—C54	−67.9 (3)	O5—C35—C36—C37	−171.7 (3)
C29—Sn2—N4—C54	78.7 (3)	N3—C36—C37—C38	2.2 (5)
N3—Sn2—N4—C54	−2.9 (4)	C35—C36—C37—C38	−177.6 (3)
O1—Sn1—C2—C3	−8.0 (3)	C36—C37—C38—C39	−0.5 (5)
C1—Sn1—C2—C3	−155.3 (3)	C37—C38—C39—C44	−1.2 (5)
O3—Sn1—C2—C3	70.3 (3)	C37—C38—C39—C40	178.6 (3)
N1—Sn1—C2—C3	−78.3 (3)	C44—C39—C40—C41	−3.3 (5)
N2—Sn1—C2—C3	137.4 (3)	C38—C39—C40—C41	177.0 (3)
O1—Sn1—C2—C7	170.6 (3)	C39—C40—C41—C42	2.7 (5)
C1—Sn1—C2—C7	23.3 (4)	C40—C41—C42—C43	−1.3 (5)
O3—Sn1—C2—C7	−111.1 (3)	C41—C42—C43—C44	0.6 (5)
N1—Sn1—C2—C7	100.3 (3)	C36—N3—C44—C43	179.3 (3)
N2—Sn1—C2—C7	−44.0 (3)	Sn2—N3—C44—C43	7.9 (4)
C7—C2—C3—C4	2.0 (5)	C36—N3—C44—C39	0.1 (5)
Sn1—C2—C3—C4	−179.3 (3)	Sn2—N3—C44—C39	−171.2 (2)
C2—C3—C4—C5	−0.5 (6)	C42—C43—C44—N3	179.7 (3)
C3—C4—C5—C6	−2.4 (6)	C42—C43—C44—C39	−1.2 (5)
C4—C5—C6—C7	3.7 (6)	C40—C39—C44—N3	−178.4 (3)
C5—C6—C7—C2	−2.2 (6)	C38—C39—C44—N3	1.4 (5)
C3—C2—C7—C6	−0.7 (5)	C40—C39—C44—C43	2.5 (5)
Sn1—C2—C7—C6	−179.3 (3)	C38—C39—C44—C43	−177.7 (3)
Sn1—O1—C8—O2	−177.3 (2)	Sn2—O7—C45—O8	−173.4 (2)
Sn1—O1—C8—C9	3.2 (4)	Sn2—O7—C45—C46	6.3 (4)
C17—N1—C9—C10	−2.6 (5)	C54—N4—C46—C47	−1.2 (5)
Sn1—N1—C9—C10	172.2 (3)	Sn2—N4—C46—C47	177.3 (3)
C17—N1—C9—C8	176.8 (3)	C54—N4—C46—C45	178.8 (3)
Sn1—N1—C9—C8	−8.4 (3)	Sn2—N4—C46—C45	−2.7 (3)
O2—C8—C9—N1	−174.8 (3)	O8—C45—C46—N4	178.5 (3)
O1—C8—C9—N1	4.6 (4)	O7—C45—C46—N4	−1.2 (5)
O2—C8—C9—C10	4.6 (5)	O8—C45—C46—C47	−1.4 (5)
O1—C8—C9—C10	−175.9 (3)	O7—C45—C46—C47	178.9 (3)
N1—C9—C10—C11	1.3 (5)	N4—C46—C47—C48	−0.2 (5)
C8—C9—C10—C11	−178.1 (3)	C45—C46—C47—C48	179.8 (3)
C9—C10—C11—C12	1.5 (5)	C46—C47—C48—C49	1.2 (5)
C10—C11—C12—C17	−2.8 (5)	C47—C48—C49—C50	−179.9 (3)
C10—C11—C12—C13	177.2 (3)	C47—C48—C49—C54	−0.8 (5)
C11—C12—C13—C14	177.0 (3)	C48—C49—C50—C51	−178.6 (3)
C17—C12—C13—C14	−3.0 (5)	C54—C49—C50—C51	2.4 (5)
C12—C13—C14—C15	2.0 (5)	C49—C50—C51—C52	−0.7 (5)
C13—C14—C15—C16	−0.2 (5)	C50—C51—C52—C53	−0.3 (6)
C14—C15—C16—C17	−0.6 (5)	C51—C52—C53—C54	−0.5 (6)
C9—N1—C17—C16	−179.5 (3)	C46—N4—C54—C53	−176.7 (3)
Sn1—N1—C17—C16	6.8 (4)	Sn2—N4—C54—C53	5.2 (5)
C9—N1—C17—C12	1.2 (5)	C46—N4—C54—C49	1.6 (5)
Sn1—N1—C17—C12	−172.4 (2)	Sn2—N4—C54—C49	−176.5 (2)
C15—C16—C17—N1	−179.7 (3)	C52—C53—C54—N4	−179.3 (3)
C15—C16—C17—C12	−0.4 (5)	C52—C53—C54—C49	2.3 (5)
C11—C12—C17—N1	1.4 (5)	C50—C49—C54—N4	178.5 (3)
C13—C12—C17—N1	−178.6 (3)	C48—C49—C54—N4	−0.6 (5)
C11—C12—C17—C16	−177.8 (3)	C50—C49—C54—C53	−3.2 (5)
C13—C12—C17—C16	2.2 (5)	C48—C49—C54—C53	177.7 (3)

### Hydrogen-bond geometry (Å, °)

**Table:**

*D*—H···*A*	*D*—H	H···*A*	*D*···*A*	*D*—H···*A*
O1w—H1w1···O2	0.84	2.09	2.914 (4)	167
O1w—H1w2···O8	0.84	1.95	2.777 (4)	167
O2w—H2w1···O4	0.84	2.08	2.911 (4)	171
O2w—H2w2···O6^i^	0.84	2.07	2.898 (4)	171

Symmetry codes: (i) *x*−1, *y*, *z*−1.
